# Ovariotomy Case

**Published:** 1872-07

**Authors:** Robt. Battey

**Affiliations:** Rome, Ga.


THE GEORGIA

### MEDICAL COMPANION:

A MONTHLY ADVISER.

VOL. 2. ATLANTA, GA., JULY, 1872. No. 7.

### PART I.

### Original Communications and Special Selections.

### OVARIOTOMY CASE.

By ROBT. BATTEY, M. D.. OF ROME, GA. *Read before the Georgia Medical Association.*

MRS. H.—aged 38, married, had one child—noticed 14 years previously a small lump deep down in the right illiac fossa. This tumor slowly increased in size, until the distension was more than is ordinarily presented at *term.* In the fall of 1864 she was tapped by Prof. B. W. Avent, of Memphis, who obtained two quarts of fluid, with considerable relief to the more urgent symptoms. Four weeks afterward Dr. Avent tapped a second time, drawing one quart. On the second day following this she aborted at about six months.

She came first under my care in 1865 at Rome, when she was occasionally troubled with attacks of severe and protracted neuralgia, caused by the mechanical pressure. In the following year I visited her in Chattooga county, and examined her with a view to operative interference. A cyst of the right ovary was diagnosed, and the uterus found to be healthy. Although there was great enlargement of the abdomen, and she still suffered occasionally from neuralgia, the state of general health was fair. Of a lively and naturally cheerful disposition, she was fairly enjoying life. She often rode on horseback for miles, and with comparative comfort. She was advised to content herself with her then state of health and comfort, and was promised an operation when decided symptoms of decline should call for it.

In the spring of 1869 the general health had given way, and her sufferings had rendered life no longer enjoyable. She had long contemplated an operation, and now earnestly desired it. I therefore visited her at Setohatchee, Ala.

May 20th, 1869, at 3 p. m., she was chloroformed and placed upon the operating table. Drs. Pritchett, Muschatt and Samply, of Haynesville, and Drs. Sanderson and McLain, of Setohatchee, were present by invitation, and assisting. An incision two and a half inches in length was made in the medium line. There being no parietal adhesions, the tumor was tapped with a large trochar, not especially adapted to such uses ; a thick white fluid, strongly reminding one of buttermilk, flowed tardily forth, and much time was wasted in efforts to discharge the cyst through the canula. A portion of the cyst-wall was now drawn out of the abdomen, and freely slit open to discharge the fluid ; a large mass of white adipose material strongly resembling "cotton seed butter" was drawn out, having much long hair intermingled with it. So striking were these resemblances that remarks were instinctively uttered by the bystanders—"Why, Doctor, you are getting butter-milk," and "There is your butter all churned ready for taking up." I did, in fact, actually "take it up," as the phrase is, by "gathering the little masses together, which loosely adhered on contact, as in a churn, and "working out the buttermilk," obtained, after the operation, near two pounds of good, solid butter, which, but for the numerous hairs which permeated it in every direction, seemed fit to be placed upon the breakfast table. The unctuous feeling in the hands was not that of lard or fat or tallow, but distinctly the impression made by freshly-churned butter—and numerous smaller fragments floated half-concealed, upon the residual buttermilk.

The cyst being emptied of its contents was gradually drawn out through the small abdominal incision ; a very firm omental adhesion was severed by *ecrasement ;* the uterus and left ovary were brought into view and found to be healthy ; a considerable little mass of the buttery material, which had accidentally escaped into the cavity of the pelvis, was removed; the pedicle was transfixed by a needle carrying a double silver wire, the wire secured upon the two halves, and severed slowly below, by the ecraseur, with a view of dropping the pedicle back into the pelvis. There was no bleeding, but as the action of the ecraseur chain had loosened the hold of the wires, it was deemed prudent to secure the pedicle in two portions with silk, and "pocket it" between the two lips of the incision held by hare-lip pins, as practiced by Dr. Storer, of Boston, in order that it might afterwards be readily reached in case of hemorrhage.

The cyst, when distended, measured thirty-six inches in its long circumference, and was estimated, as nearly as could be attained, to weigh thirty pounds with its contents. Of the contained materials, besides those already named, were many teeth, both loose and adherent, and several fragments of bone. A canine tooth was large and very perfectly formed, and one bony fragment was distinctly fashioned for the petrous portion of the temporal with part of the auditory canal.

6 p. m.—Pulse 84, patient comfortable and quite cheerful.

21*st May.*—Pulse 96 a. m., and 80 p. m.; doing finely, no treatment, simply nursed.

22*d May.*—Pulse 84 a. m., 80 p. m., had a good day—says she "feels very well"—adhesion is good throughout the abdominal wound.

23*d May.*—Pulse 80 a. m., 84 p. m., is doing finely.

24*th May*—Pulse 84 a. m., very comfortable—removed the pins and superficial sutures, lips of the wound are firmly adherent, walls of the abdomen flat and loose, good appetite, rests comfortably, is cheerful and happy. I returned to my home leaving her in the care of her husband, Dr. H., who reported her condition to me from day to day by mail. From these reports the following record is made up:

24th p. m.—Pulse 110, slight fever, restless, required laudanum enema—slight oozing of blood from points where the pins were removed.

25*th Mag.*—Pulse 100 a. m., 94 p. m., resting well—did not require any anodyne. The pedicle makes decided traction upon the abdominal walls, drawing the wound strongly inwards.

*26th May.*—Pulse a. m. 94, p. m. 84—required opiate in morning, is free of fever and quite cheerful.

*27th May.*—Pulse a. m. 90, p. m. 96—slept all night—a little fever in p. m., bowels slightly tympanitic.

*28th May.*—Pulse 88 and 90—no opiate to-day, two doses of quinine.

*29th May.*—Pulse 86 and 88—doing well.

*30th May.*—Pulse 90 and 90—rested badly, rheumatism in shoulder, bowels not yet moved, appetite good, wound healthy, slight traction does not move the ligature.

*31st May.*—Pulse 90 and 86—rested badly, rheumatism shoulder and chest.

*1st June.*—Pulse 92 and 96—still complaining of rheumatism, no evacuation of the bowels yet, abdomen tympanitic, mild enema morning and evening did not move the bowels—menses appeared to-day, prematurely—ligature has not come away yet.

*2d June.*—Pulse 88 and 82—More feeble, otherwise, doing well, two enemata, no alvine evacuation yet, less appetite.

*3d June.*—Pulse 82 and 86—enema, bowels moved freely—rheumatism still, menses normal, seems quite feeble, but otherwise doing well, very well.

*4th June.*—Pulse 80 and 94—menses ceasing, appetite good.

*5th June.*—Pulse 85 and 90—bowels moving heathfully, wound is healing nicely—ligature not away yet.

*6th June*—Pulse 86 and 100, little fever again, no appetite—takes 10 drops tincture iron tri-daily.

*7th June*—Pulse 86 and 88, appetite better, gaining strength—had 9 grains quinine.

*8th June*—Pulse 84 and 86, one thread of ligature came away, very comfortable.

*10th June*—Pulse normal, gaining, sits up—impatient for the other ligatures to come away.

*12th June*—Gets up and dresses without assistance—another thread came away.

*19th June*—Progressing admirably—Dr. H. says, "as gay as a cricket"—another ligature removed.

*3d July*—" She is attending daily to her domestic affairs."

*22d July*—Mrs. H. sends word " she is well now." The ligatures are all away and the wound is healed.

Mrs. H. has enjoyed excellent health up to the present time. She has been regular and healthy in her menstrual function, has had no miscarriage, but I regret to say, is not yet " blest in her household."

